# A case study of Ramsay Hunt Syndrome in conjunction with cranial polyneuritis

**DOI:** 10.1097/MD.0000000000008833

**Published:** 2017-11-27

**Authors:** Ru-Wen Zheng, Di Liu, Tay E. Eric, Yan-Zhe Ning, Lu-Lu Chen, Hui Hu, Yi Ren

**Affiliations:** a Department of Acupuncture and Moxibustion, Dongfang Hospital, The Second Affiliated Hospital of Beijing University of Chinese Medicine, Beijing, China; b Singapore Chung Hwa Medical Institution, Singapore; c Beijing Anding Hospital, Affiliated to Capital Medical University; d Department of Neurology, Dongzhimen Hospital, The First Affiliated Hospital of Beijing University of Chinese Medicine, Beijing, China.

**Keywords:** herpes zoster, misdiagnosis, Ramsay Hunt syndrome, vertigo

## Abstract

**Rationale:**

Ramsay Hunt syndrome in conjunction with cranial polyneuritis is not extensively documented, and is very easily misdiagnosed.

**Patient concerns:**

A case of a 53-year-old male with Ramsay Hunt syndrome in conjunction with cranial polyneuritis is presented with early symptoms of vertigo, cephalalgia, and facial palsy, followed by zoster oticus 10 days later.

**Diagnoses:**

Diagnosis was challenging as this condition presents with multiple neuropathies, and attempting to diagnose based on clinical symptoms was often misleading. Polymerase chain reaction can be used to test for presence of the virus in the cerebrospinal fluid, followed by targeted drug therapy.

**Interventions:**

Acupuncture, in conjunction with fire cupping, bloodletting around the afflicted region on the face, as well as oral consumption of herbal medicine and vitamins for nerve nourishment was given to treat this disease.

**Outcomes:**

Due to misdiagnosis resulting in delayed treatment, peripheral facial paralysis was left as the main sequelae, while other symptoms responded quickly to treatment. After a 6-month follow-up, facial palsy was still present.

**Lessons:**

Considering that targeted antiviral therapy can be used to increase the effectiveness of treatment, early diagnosis, and timely use of medication is critical.

## 1 Introduction

Ramsay Hunt syndrome was first described in 1907 by James Ramsay Hunt, and is characterized by ipsilateral paroxysmal pain deep within the ear, erythematous vesicular rash around the auricle and auditory canal, as well as peripheral facial paralysis.^[1]^ It is caused by the varicella zoster virus (VZV), and can affect the vestibular, trigeminal, and cochlear nerve resulting in various cranial neuropathies.^[2]^ The research has shown that cranial polyneuritis presents in 1.8% of all patients suffering from Ramsay Hunt syndrome.^[3]^ In such cases, accompanying symptoms such as auditory and balance impairments may be observed. These associated symptoms vary from case to case, depending on the physical condition of the patient and route of infection, and may appear before or after the main symptoms of Ramsay Hunt syndrome. This severely complicates the diagnosis of the condition, delaying the necessary treatments. Early diagnosis and timely medical intervention is vital for Ramsay-Hunt syndrome patients due to the low spontaneous recovery rate and potential sequelae. We encountered a clinical case study of a patient initially presenting with facial palsy accompanied by intense vertigo and cephalalgia, followed by eruption of erythematous vesicular rash around the ear 10 days later. In this case, it was not possible to make an accurate diagnosis in the early stage of the disease.

## 2 Case report

A 53-year-old male was admitted to our hospital with left-sided facial drooping, headache, and dizziness for 6 days. Clinical history: during the night on July 26, 2016, the patient felt a sudden pain behind the left ear, extending to the left side of the head. The next morning, he awoke to find his eyelid drooping and that he was unable to fully close his left eyelid, accompanied by inability to wrinkle forehead, inability to frown, drooping at the corner of his mouth, and inability to blow out his cheeks. In addition to the onset of dizziness, the pain behind his left ear and on the left side of his head had also worsened. A cranial computed tomography (CT) scan conducted in another hospital found no abnormalities. A subsequent checkup by their department of otorhinolaryngology did not find any skin lesions in the auricle or auditory canal. The initial diagnosis was Bell palsy, and a treatment course of acupuncture and vitamins for nerve nourishment was given, but there was no improvement. Symptoms upon admission to our hospital: left eyelid ptosis and inability to close, flattening of facial creases as well as inability to frown, drooping at the corner of his mouth, and inability to blow out his cheeks. Pain was experienced behind the left ear, along with vertigo, cephalalgia, tinnitus, xerostomia, dysgeusia, and scattered oral ulcerations. Medical history: 35-year history of hypertension, 15-year history of type 2 diabetes mellitus, and radiofrequency ablation for prolapse of lumbar intervertebral disc 25 days before. Physical examination findings included pain in the left mastoid bone upon palpation, and disappearance of left side forehead creases and hypophasis in left eyes. Diagnosis: Ramsay Hunt syndrome.

Treatment process: on August 2, 2016, vertigo and cephalalgia worsened, and patient was unable to stand up. His blood pressure ranged between 160–170/80–90 mm Hg. An emergency magnetic resonance imaging (MRI) revealed small ischemic focus on both sides of the parietal cortex. A routine blood test did not detect any abnormalities. The patient was placed on an intravenous drip to improve the blood circulation and nourish the nerves. On August 3, 2016, the patient's temperature rose and fluctuated between 36.8 and 37.9°C. A second routine blood test showed WBC: 11.91 × 10^9^/L; NE%: 77.5%; and LY%: 15.5%. Suspecting an infection, cefuroxime axetil (0.25 g, bid) was enterally administered. On that same day, vertigo and cephalalgia worsened, the patient experienced severe dizzy spells, was unable to stand up and vomited 3 times. A lumbar puncture to check the cerebrospinal fluid (CSF) did not find any abnormalities. On August 5, an ENT consultation found scattered vesicular eruptions in the left auricle and auditory canal. The vestibular autorotation test revealed spontaneous horizontal nystagmus in the nonaffected direction. Diagnosis: herpes zoster oticus and vestibular neuritis. Treatment consisted of cefotaxime sodium, acyclovir IV infusion, combined with an externally applied topical cream and prednisone acetate tablets 30 mg/d for 3 days, followed by 20 mg/d for next 3 days, and 10 mg/d for further 3 days. Traditional Chinese medicine treatments consisted of acupuncture, in conjunction with fire cupping, bloodletting around the afflicted region on the face, as well as oral consumption of herbal medicine. Vesicular rashes around the ear gradually subsided, and there was significant reduction in vertigo and tinnitus. However, residual symptoms such as facial drooping remained, and the patient was instructed to follow up with acupuncture treatment in outpatient clinic.

## 3 Discussion

In this case, the patient initially presented with vertigo and cephalalgia, followed by appearance of peripheral facial paralysis while eruption of vesicular rashes around the auricle and auditory canal appeared last. In the case of Ramsay Hunt syndrome in conjunction with cranial polyneuritis, as the characteristic symptoms were preceded by certain neuropathy symptoms, medical personnel were unable to make an accurate diagnosis at the beginning. A discussion of the diagnosis and treatment in this case study is as follows.

With initially symptoms of vertigo and cephalalgia, 35-year history of hypertension, and the absence of any abnormalities in the cranial CT scan, the vertigo was considered to be linked with the patient's fluctuating blood pressure. As the vertigo continued for approximately a week and became progressively more severe, with the gradual appearance of symptoms such as quadriparesis and abasia, it was suspected that the various symptoms could be due to posterior circulation ischemia. A head MRI scan subsequently revealed the presence of ischemic focus. Academics postulated that the virus spread via Cranial nerve V and its ganglia, causing inflammatory swelling in the canal and impaired blood flow. Accordingly, the function of larger vasculatures in the region can also be affected. Cranial nerves III, IV, V, and VI receive their blood supply from the internal carotid artery, whereas cranial nerves V, VII, IX, X, XI, and XII are supplied by the external carotid artery. Through high-resolution MRI, it was found that VZV affected the internal carotid artery by causing narrowing of the vasculature and thickening of the vascular wall. The main areas affected were the distal ends of the internal carotid artery, proximal ends of M1 segment of the middle cerebral artery, as well as A1 segment of the anterior cerebral artery and proximal end of P1 segment of the posterior cerebral artery.^[4]^

As the possibility of a severe cerebrovascular condition was eliminated, the worsening vertigo and cephalalgia in conjunction with nausea and vomiting, elevated leukocyte and neutrophil count could have been an indicator of meningitis. A lumbar puncture to check for the presence of bacterial or viral infection tested negative. As the VZV lay dormant in the geniculate ganglion, reactivation of the virus could damage the cranial nerves and lead to diverse symptoms resembling that of central nervous system conditions such as myelopathy, encephalitis, meningitis, cerebrovascular diseases, etc.^[5]^ If the patient does not present with characteristic vesicular rashes, it will be difficult to make an accurate diagnosis. Polymerase chain reaction detection of VZV DNA in the CSF can be conducted followed by targeted antiviral treatment. Early diagnosis and timely medical intervention is of utmost importance in the treatment of this disease.

Besides presenting with vesicular rashes around the left auricle and auditory canal, the patient also had other symptoms such as facial palsy, tinnitus, and vertigo. The above symptoms correspond to cranial nerves V, VII, and VIII. As the cerebral hemorrhage, cerebral infarction, and meningitis had been already ruled out, the abovementioned symptoms would be caused by the associated cranial nerves following an infection by the VZV.

Ramsay Hunt syndrome is due to the dormant VZV in the geniculate ganglions. Academics have proposed that following the reactivation of the virus, the synapse is one of the means through which the virus spreads through the nervous system, thus affecting the cranial nerves.^[6]^ Hence in the early stages of the disease when symptoms such as vesicular rashes and facial palsy have yet to manifest, multiple cranial neuropathies could lead to various initial symptoms and possible misdiagnosis. For instance, in the case of throat lesions or ulcers where the upper respiratory tract symptoms are more prominent than the otological symptoms, it is often misdiagnosed as an upper respiratory tract infection.^[7]^ If facial pain is significant and presents prior to the onset of facial palsy, the case is often misdiagnosed for trigeminal neuralgia. Ramsay Hunt syndrome is 1 common cause of peripheral facial nerve paralysis, but if the facial palsy symptoms present before the appearance of vesicular rashes around the ear, it is commonly misdiagnosed as Bell palsy instead. Patients with Ramsay Hunt syndrome often suffer from more severe facial neuropathies compared with Bell palsy, and often are left with more serious sequelae. Thus, if peripheral facial paralysis presents along with nervous system disorders such as auditory impairment, there is sufficient cause to suspect Ramsay Hunt syndrome. Timely administration of sufficient antiviral medication combined with high-dose steroid treatment is effective in stimulating recovery.^[8]^

In this case, vertigo, cephalalgia, and pain behind the ear were the initial presenting symptoms, followed by onset of facial palsy, and herpes zoster oticus appeared last. In hence, Ramsay Hunt syndrome in conjunction with cranial polyneuritis is considered. When a person is infected with the VZV, if antiviral medications are not administered in time, there is a risk of cranial nerves being infected, as well as the possibility of intracranial vascular pathologies, resulting in suffering cerebral ischemia, cerebral apoplexy, progressive cognitive decline, etc. The patient was misdiagnosed during the early stages and did not receive the necessary antiviral treatment in time, which caused the patient to get worse.

## 4 Conclusion

Ramsay Hunt syndrome is not a rare disease; however, due to the virus spreading through the nervous system resulting in a diverse range of cranial neuropathies and corresponding symptoms that mask the characteristic symptoms of Ramsay Hunt syndrome, misdiagnosis commonly occurs. At the same time, clinical researches found that timely therapeutic intervention using acupuncture and medication was the key to achieving a better and more complete recovery. In hence, there is a need for more emphasis on the differential diagnosis for Ramsay Hunt syndrome, to achieve an early and accurate diagnosis.
